# The Numerical Study of the Hemodynamic Characteristics in the Patient-Specific Intracranial Aneurysms before and after Surgery

**DOI:** 10.1155/2016/4384508

**Published:** 2016-05-05

**Authors:** Jun Soo Byun, Sun-Young Choi, Taewon Seo

**Affiliations:** ^1^Department of Radiology, Chung-Ang University Hospital, No. 102, Heukseok-ro, Dongjak-gu, Seoul 06973, Republic of Korea; ^2^Department of Radiology and Medical Research Institute, School of Medicine, Ewha Womans University, No. 1071, Anyangcheon-ro, Yangcheon-gu, Seoul 07985, Republic of Korea; ^3^Department of Mechanical and Automotive Engineering, Andong National University, No. 1375, Gyeongdong-ro, Andong-si, Gyeongsangbuk-do 36729, Republic of Korea

## Abstract

The patient-specific pre- and postsurgery cerebral arterial geometries in the study were reconstructed from computed tomography angiography (CTA). Three-dimensional computational fluid dynamics models were used to investigate the hemodynamic phenomena in the cerebral arteries before and after surgery of the aneurysm under realistic conditions. CFD simulations for laminar flow of incompressible Newtonian fluid were conducted by using commercial software, ANSYS v15, with the rigid vascular wall assumption. The study found that the flow patterns with the complex vortical structures inside the aneurysm were similar. We also found that the inflow jet streams were coming strongly in aneurysm sac in the presurgery models, while the flow patterns in postsurgery models were quite different from those in presurgery models. The average wall shear stress after surgery for model 1 was approximately three times greater than that before surgery, while it was about twenty times greater for model 2. The area of low WSS in the daughter saccular aneurysm region in model 2 is associated with aneurysm rupture. Thus the distribution of WSS in aneurysm region provides useful prediction for the risk of aneurysm rupture.

## 1. Introduction

A cerebral aneurysm is a disease of the vascular wall causing pathological dilatations that frequently occur at the arterial bifurcations in the circle of Willis. The most serious consequence of cerebral aneurysm is the subarachnoid hemorrhage caused by the aneurysm rupture [1–4]. Since patient with unruptured aneurysm does not show any symptoms in daily life, the detection of the intracranial aneurysm and its prevention and treatment are very difficult. The recent enhancement in the technology of the imaging diagnosis systems, such as computed tomography angiography (CTA) and magnetic resonance angiography (MRA), has facilitated the detection of the unruptured aneurysm, therefore allowing for the early treatment to prevent subarachnoid hemorrhage [5, 6].

There is a correlation between the size of an aneurysm and the likelihood of its rupture; for example, the larger the aneurysm is, the more likely it is that the rupture will occur [7, 8]. However, because the size and the shape of an aneurysm vary with patients, evaluating the risk of rupture based on these factors is limited [9, 10]. Since the prognosis of subarachnoid hemorrhage is still difficult to predict, there has been an increase in prophylactic surgery. Current treatment options include surgical clipping of the aneurysm neck and endovascular coiling, but these procedures have the risks and complications such as stroke and aneurysm rupture. So far, aneurysm size and location and multiplicity and patient's age, family history, and medical history have been considered the predominant factor to determine whether the surgery should be conducted or not. If the diameter of an aneurysm is greater than 5 mm, a surgery is generally recommended [11, 12]. According to the study of Weir et al. [10] the average size of aneurysm is 8.7 mm, in which a rupture could occur.

Although the mechanisms of aneurysm initiation, growth, and rupture have not been completely understood yet, the interaction of hemodynamic forces on the vascular wall is generally accepted as the risk factor. The process of cerebral aneurysm formation, progression, and rupture is believed to be related to the hemodynamics. Endothelial cells can have the ability to detect the fluid wall shear stress (WSS) and consequently adapt their spatial organization in the hemodynamic environment. Previous studies [8, 12] have shown that a uniform shear stress field causes the endothelial cells to stretch and align in the flow direction. On the other hand, the low and oscillatory shear stress fields cause the irregular shape and the loss of the cells' orientation. Thus, the low and oscillatory patterns of the WSS promote the intimal wall thickening as well as the atherosclerosis [1, 4, 11]. Many* in vitro* and numerical observations [13–15] have reported the effects of WSS on the development, growth, and rupture of intracranial aneurysm.

According to the high flow theory [4], the elevated WSS can initiate the wall remodeling and weaken the wall due to an abnormal shear stress field. On the contrary, the low flow theory explains that the low blood flow in the aneurysm can result in the blood stagnation in the dome. The dysfunction of the aggregation of RBC (red blood cell), the accumulation and the adhesion of platelets, and leukocytes along the intimal surface are induced by the blood stagnation. This process may cause inflammation and leads the aneurysm wall to weaken and ultimately encounter an aneurysm rupture.

The cerebral aneurysm typically occurs at the outer curvatures, bifurcations, and branching points of the cerebral arteries. Studies indicate that the blood flows in these regions are characterized by complex flow patterns, such as the strong secondary flows and flow stagnation [1, 2, 16]. In particular, the distributions of WSS acting on the arterial wall periodically increase and decrease in magnitude. The larger the aspect ratio of aneurysm, the smaller the WSS in the dome. The low WSS along endothelial surface coagulates the blood cells, including the red blood cells and platelets, and leads to endothelial cell dysfunction and loss. It is predicted that the low WSS can lead to an aneurysm rupture by the endothelial cell damage, thrombus, and inflammation on the aneurysm wall.

Since the hemodynamic forces act on aneurysm, the hemodynamic factors, such as WSS, pressure on the aneurysm, and the secondary flow patterns, are significant parameters of aneurysm rupture. These factors are strongly dependent not only on the geometry of the arteries but also on the shape of aneurysm. The aim of this study is to compare the hemodynamic phenomena in the cerebral arteries before and after surgery of the aneurysm under realistic conditions. To investigate the hemodynamic characteristics in brain aneurysm, the patient-specific pre- and postsurgery cerebral arterial geometries were reconstructed from medical data and blood flow has been simulated using computational fluid dynamics (CFD).

## 2. Formulation of the Problem

### 2.1. Vascular Models Reconstruction

**Figure 1:**
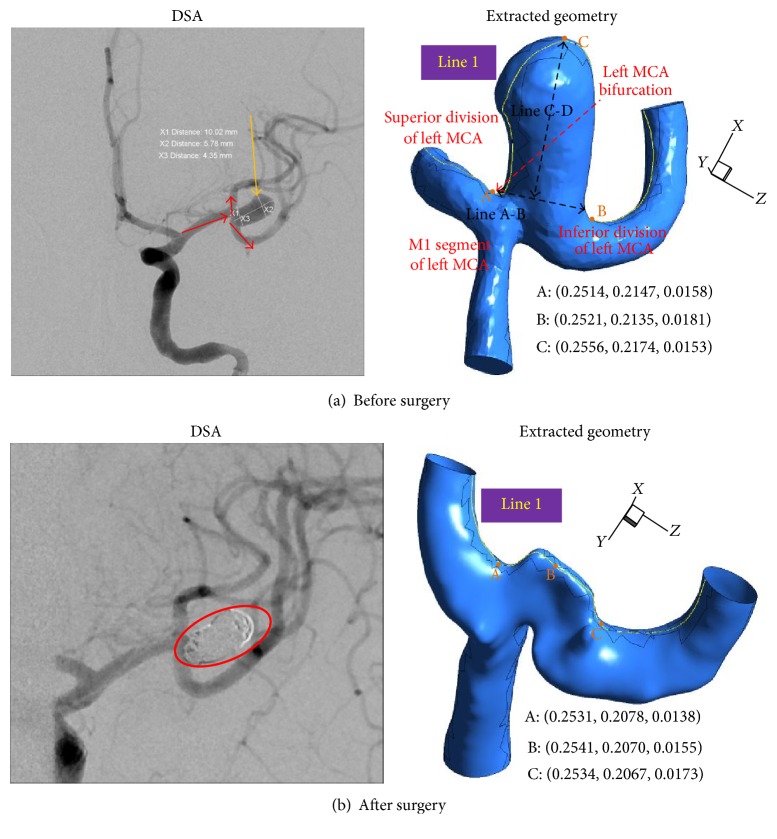
Angiography and extracted pre- and postsurgery cerebral arterial geometries for model 1.

**Figure 2:**
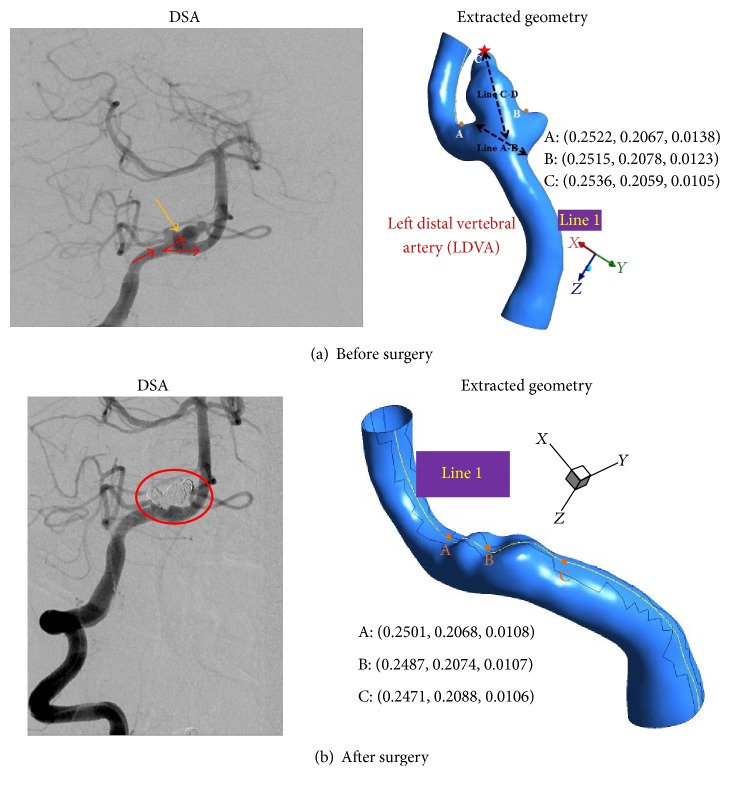
Angiography and extracted pre- and postsurgery cerebral arterial geometries for model 2.

All medical data in the study were acquired via DSA (Digital Subtraction Angiography, Axiom Artis, Siemens Medical System, Germany) from two patients in Chung-Ang University Hospital. Starting from the medical images, the suitable geometry models were generated in order to conduct the desired blood flow simulations. Images produced from DSA are volume data consisting of 101 slices of 2,172 × 1,860 pixels' images. MIMICS software imported these DICOM (Digital Imaging and Communications in Medicine) image files to reconstruct cerebral aneurysm models. The slice thickness of the scans is 9.11 *μ*m. After loading the image files into MIMICS, the user can see images in the axial (*XY* plane), coronal (*XZ*), and sagittal (*YZ*) directions. The most important first step to convert the image files into 3D model is a segmentation process. During segmentation the user creates 3D model based on the gray values within these DICOM files using thresholding. The user can apply the “region growing” tool to remove any floating pixels in the images and to separate the bone from the blood vessel. Finally the geometry models from the medical images coming from DSA shown in Figures 1 and 2 are obtained using the image segmentation and three-dimensional model creation, as mentioned. Both cases of models 1 and 2 were ruptured aneurysms, so these aneurysms were treated by endovascular coiling (refer to red circles in Figures 1 and 2)—also calling it endovascular surgery.

In model 1, the blood flowing from the M1 segment of left middle cerebral artery (MCA) divides at the left MCA bifurcation and flows into the superior division of left MCA and inferior division of left MCA. In contrast, model 2 shows the blood flowing straight along the left distal vertebral artery. The possible reasons for aneurysms rupture are aspect ratio, size, and location as well as the shape of the daughter sac. In model 2 there exists the daughter sac as indicated by the red star in Figure 2(a).

### 2.2. Computational Models and Simulations

The geometries generated from MIMICS are imported in ANSYS workbench to carry out the preprocessing, processing, and postprocessing. Although the blood has a Non-Newtonian behavior, it is considered a Newtonian fluid because there are no significant differences for WSS in the large arteries between Newtonian and Non-Newtonian cases [3, 15, 17]. Additionally, the Non-Newtonian effect can be negligible if the Reynolds number ranges from 100 to 850 [18]. Rigid cerebral arterial walls assumption is used to simplify the computation. Thus, the mass and momentum conservation equations for an incompressible Newtonian fluid neglecting the influence of body force, such as gravity, can be written as

(1) ∇·u→=0,∂u→∂t+u→·∇u→=−1ρ∇p+μρ∇2u→,

where $\vec{u}$, *ρ*, and *μ* denote blood velocity vector, blood density (1,050 kg/m^3^), and blood dynamic viscosity (0.0035 Pa-s), respectively.

**Figure 3:**
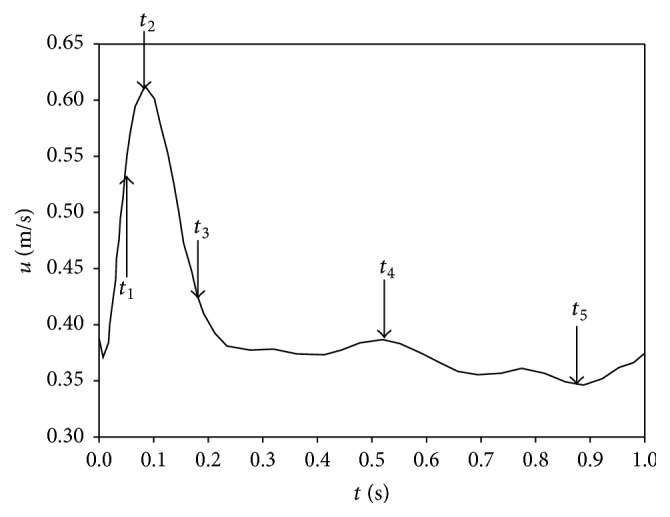
Physiological waveform of inlet velocity.

The boundary conditions were as follows: since patient-specific blood flow information was not available, a physiological velocity profile was as shown in Figure 3 at the inlet [1]. Every time step the inlet velocity can be calculated using zeroth-order interpolation method (refer to ANSYS FLUENT user's guide). The physiological outlet pressure at the iliac and the renal arteries will be different. However, we did not have any pressure information at the outlet in both the iliac and renal arteries. Thus we assumed that zero pressure at the outlet boundary was employed, and no-slip condition was applied at arterial wall surfaces. The mean inlet velocity varies between 0.35 and 0.61 m/s within 1 cardiac cycle. The Reynolds numbers at the maximal, minimal, and mean rates of blood flow were approximately 220, 124, and 154, respectively. The peak systolic flow occurred at *t* = 0.08 s as shown in Figure 3. The Womersley number, *α*, is defined by $\alpha =\left(D/2\right)\sqrt{\rho \omega /\mu }$. *ω* represents the angular frequency of the pulsatile flow and *D* is the diameter of inlet. In this simulation, the Womersley number was approximately 0.823. In Figure 3, five different times (*t*_1_, *t*_2_, *t*_3_, *t*_4_, *t*_5_) were selected to analyze the hemodynamic behaviors. The inflow at the inlet of middle cerebral artery accelerates early in the inlet velocity temporal waveform, *t*_1_ = 0.05 s, and reaches the maximum of 0.61 m/s at *t*_2_ = 0.08 s. After this point, the velocity magnitude begins to decrease and drops to 0.42 m/s at *t*_3_ = 0.18 s. Then, the blood flow slightly oscillates in the range between 0.38 (*t*_4_ = 0.52 s) and 0.35 m/s (*t*_5_ = 0.88 s).

The governing equations were solved using the commercial software package FLUENT, ANSYS 15 (ANSYS Inc., Canonsburg, PA). The QUICK scheme was used to discretize the velocity and pressure variables using the PISO (Pressure Implicit with Splitting of Operators) algorithm. The implicit time-marching first-order scheme with the time step Δ*t* = 0.01 was used for the calculations, and the maximum iterations per time step were set to 2,000.

To establish mesh independence of our numerical results, we have tested a wide range of mesh densities. For model 1 a typical mesh used in calculating the results consists of 1.56 million element cells, while for model 2 the adapted number of mesh elements is 1.87 million element cells. At this mesh density, the deviation in WSS values relative to a finer mesh is smaller than 1%. The usual mesh size, dx, is about the range of 1.74 *∗* 10^−4^~8.77*∗*10^−4^ mm.

To obtain the stable solutions, the computation performed four cardiac cycles and the result at the fourth cardiac cycle was used for the analysis in order to decrease numerical errors compared to the result at the third cardiac cycle less than 1%. The convergence tolerance for the continuity and velocity residuals was set at 10^−5^. The PCs used for simulations were based on the Intel® Core*™* i7-3770K processor with 3.5 GHz clock speed and 32 GB RAM memory running on the 64-bit Windows 7. The simulation time for the computational domains used in the study was approximately 96 CPU hours.

## 3. Results and Discussion

### 3.1. Flow Structures Comparison in the Aneurysms before and after Surgery

**Figure 4:**
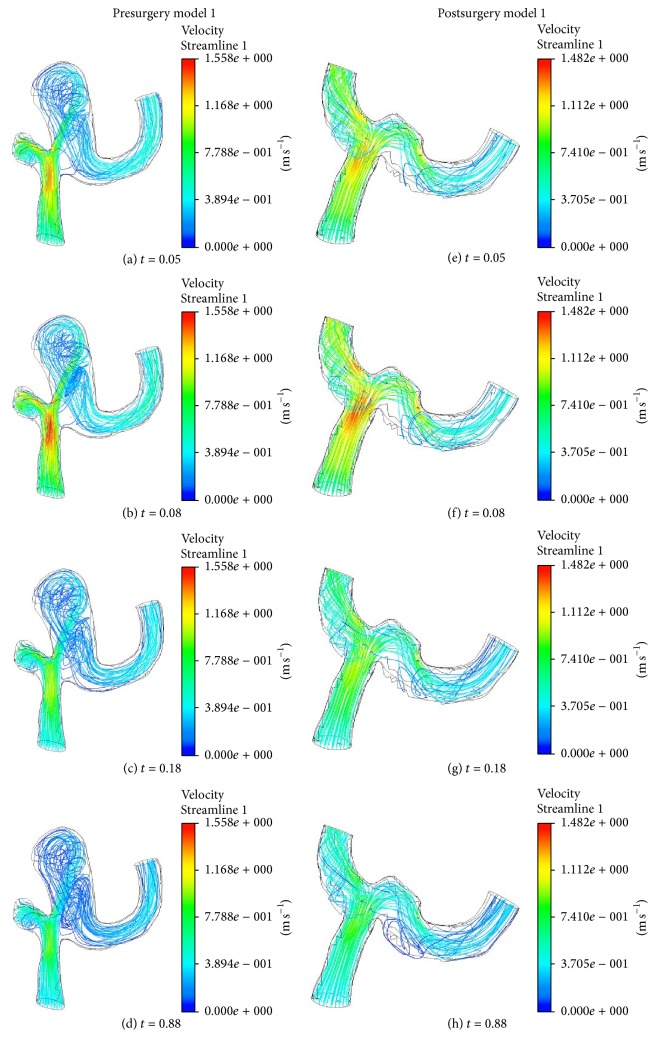
Streamlines on geometry model 1 before and after surgery at four different times.

**Figure 5:**
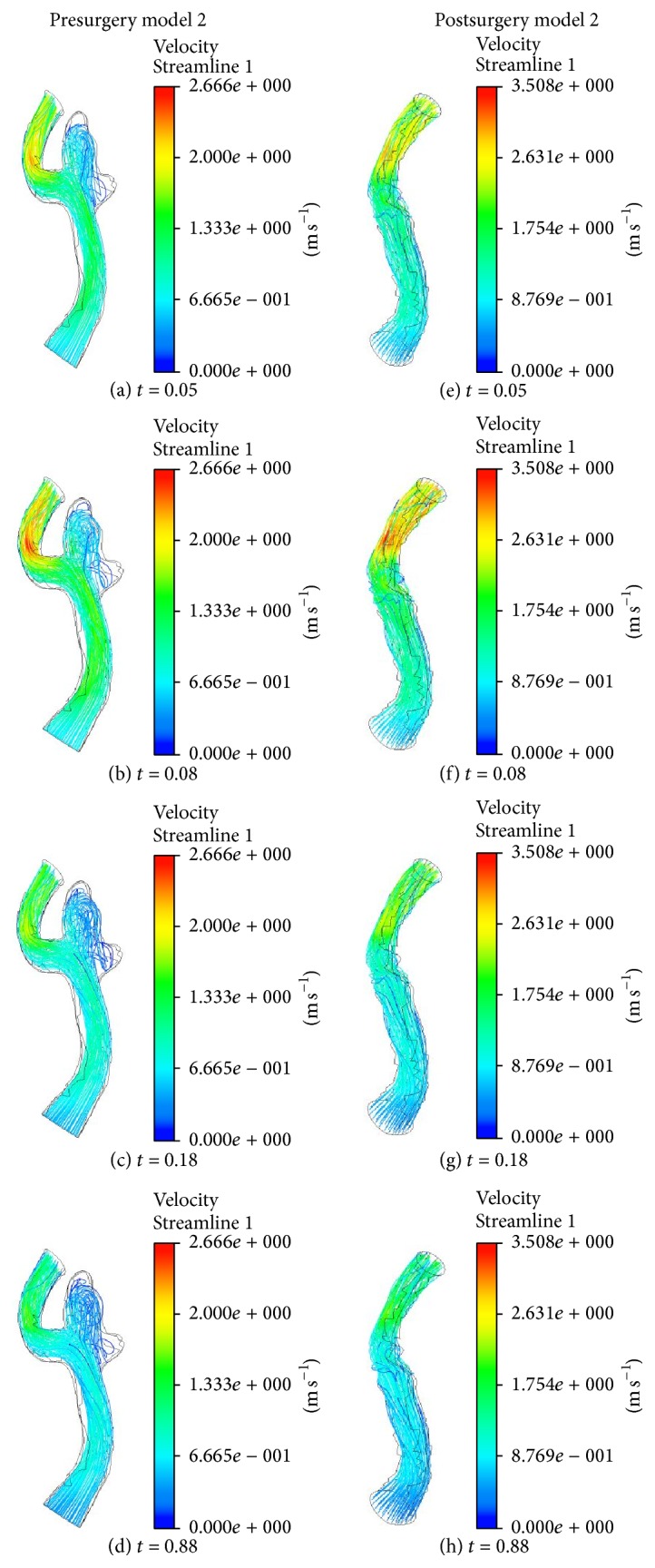
Streamlines on geometry model 2 before and after surgery at four different times.

Figures 4 and 5 show the streamlines in the blood vessel with aneurysm before and after surgery for models 1 and 2. The general characteristics of flow in the geometries of aneurysm of models 1 and 2 are complex flow patterns with multiple vortices. Unlike those of the presurgery model, the flow structures in the geometries after surgery show the simple flow patterns. At presurgery model 1, it can be seen that the blood coming from the M1 segment of left MCA hits the outer wall of the superior division of the left MCA at the left MCA bifurcation. During the accelerating and peak systole at *t* = 0.05 and 0.08 (see Figures 4(a) and 4(b)), the blood is mainly introduced to the superior division of left MCA. However, at decelerating and late diastole (see Figures 4(c) and 4(d)), blood streams spread in the whole region of aneurysm and demonstrate the formation of complex flow patterns. As a result, this leads to a helical flow structure and the recirculation moves towards the inferior division of left MCA. The jet of blood through the neck of aneurysm can impinge on the region towards the superior division of left MCA at the left MCA bifurcation.

In postsurgery model 1, as shown in Figures 4(e)~4(h), the jet flow coming from the M1 segment of left MCA can make the impact on the neck of the removed aneurysm in the left MCA bifurcation. Then the flow divides almost evenly into the superior division of left MCA and inferior division of left MCA. The size of the recirculation zone along the outer wall in the inferior division of left MCA increases as the blood velocity decreases over time. In postsurgery model 1 the incoming jet distributes evenly spread in the area of operated aneurysms, while the inflow jet impinges the small region in aneurysm sac containing the vertical structures in Figure 4.

Upon comparing the flow distributions in pre- and postsurgery model 2 in Figure 5, it can be seen that the blood smoothly flows through the left distal vertebral artery except in the aneurysm region. In presurgery model 2, the result shows that the strong helical flow is generated inside the aneurysm. For postsurgery model 2, the incoming flow has the centrifugal force due to the curvature in the inlet region and blood leads to the impact on the surgical area.

**Figure 6:**
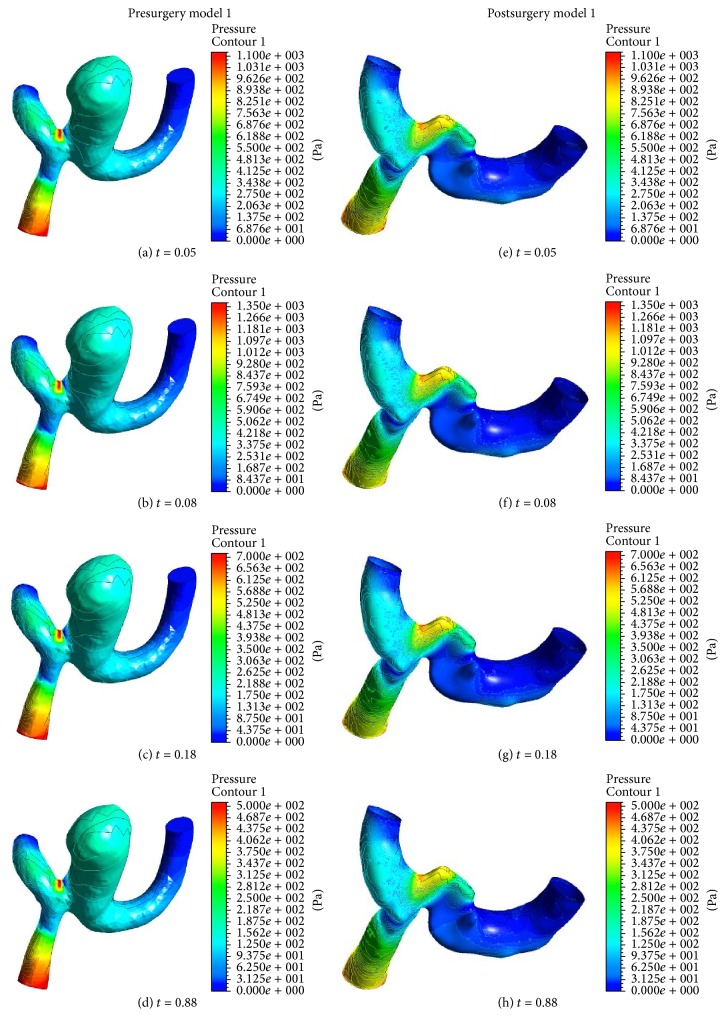
Pressure distribution on geometry model 1 before and after surgery at four different times.

**Figure 7:**
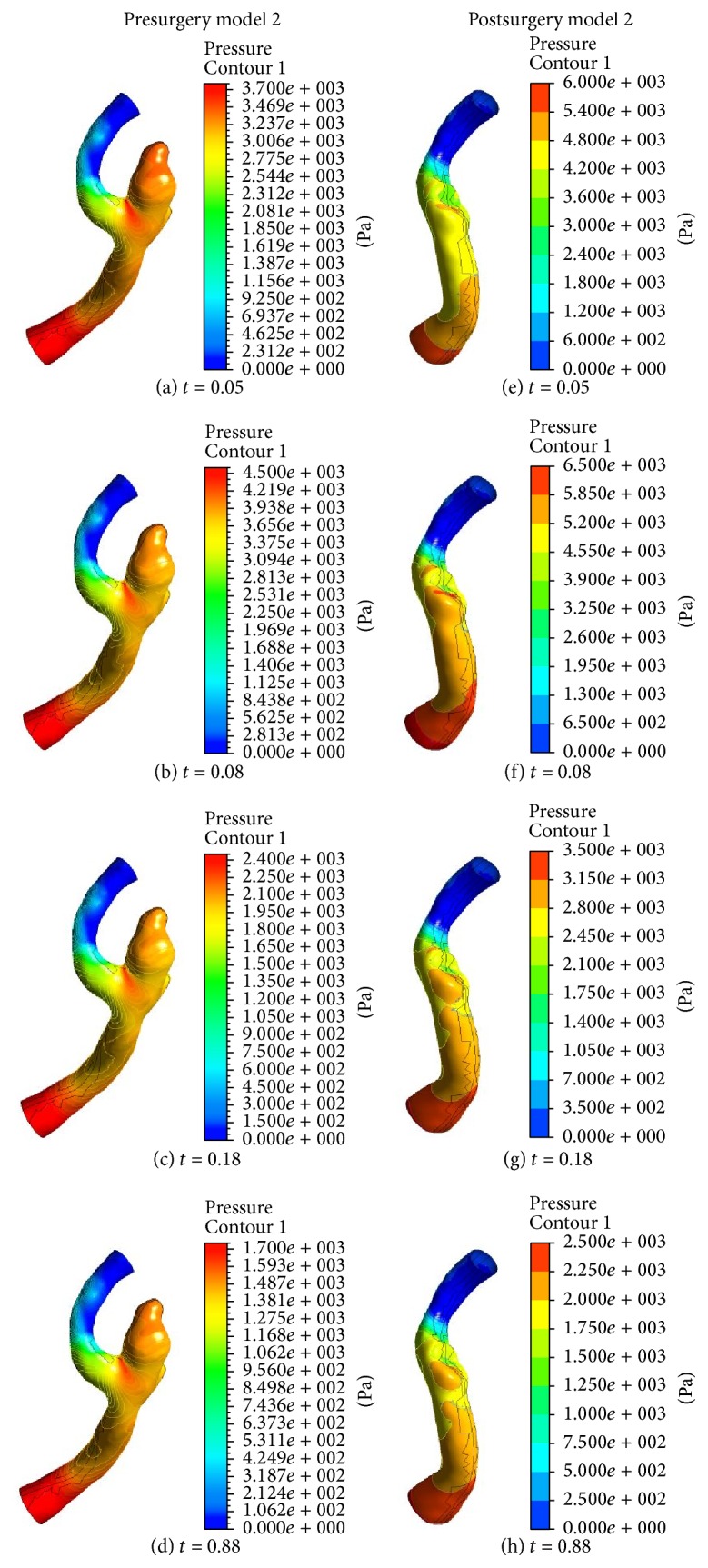
Pressure distribution on geometry model 2 before and after surgery at four different times.

Figures 6 and 7 show the results of the pressure distributions on the arterial wall in pre- and postsurgery models 1 and 2. For presurgery model 1, the highest pressure acts at the corner of the left neck of aneurysm in the left MCA bifurcation. However, the highest pressure occurred at the right side of the neck of the removed aneurysm in the inferior division of left MCA for postsurgery model 1. For model 2, the peak pressure can be seen to appear in the left region of the aneurysm neck. It is believed that the inertia increases in the region of aneurysm, while the maximum pressure acts with increasing momentum due to the strong curvature of the blood vessel.

Based on the results in models 1 and 2, we could see that the inflow jet streams in the presurgery models flew into the aneurysm sac much more strongly than those in the postsurgery models. The strong, concentrated jet streams in the presurgery models had a forceful impact on the small surfaces in the aneurysm. On the other hand, the flow patterns in the postsurgery models were quite. For example, the inflow jet streams in the postsurgery models diffused more quickly. Consequently, the jet streams hit across a wider region in the aneurysm and the impact was not as severe.

### 3.2. Wall Shear Stresses in the Aneurysms before and after Surgery

Since the hemodynamic forces such as WSS, pressure, and the flow patterns that act on the aneurysm are significant factors of aneurysm rupture, the WSS variations depend on the vessel geometry and aneurysm irregularity. Seo and Byun [15] mentioned that, by increasing the aspect ratio of aneurysm, the WSS around the dome of aneurysm gets smaller and damages endothelial cells, which results in the inflammation of the wall of aneurysm. Consequently, one can predict that an aneurysm rupture will occur.

If the sizes of aneurysms are greater than 10 mm and the aspect ratio is larger than 1.6 [10], aneurysms have a high probability of rupturing. The aspect ratio, defined as (Line C-D)/(Line A-B), of an aneurysm is 2.04 in model 1, while the aspect ratio of model 2 is 1.58. According to these criteria, model 1 is likely to rupture compared to model 2.

**Figure 8:**
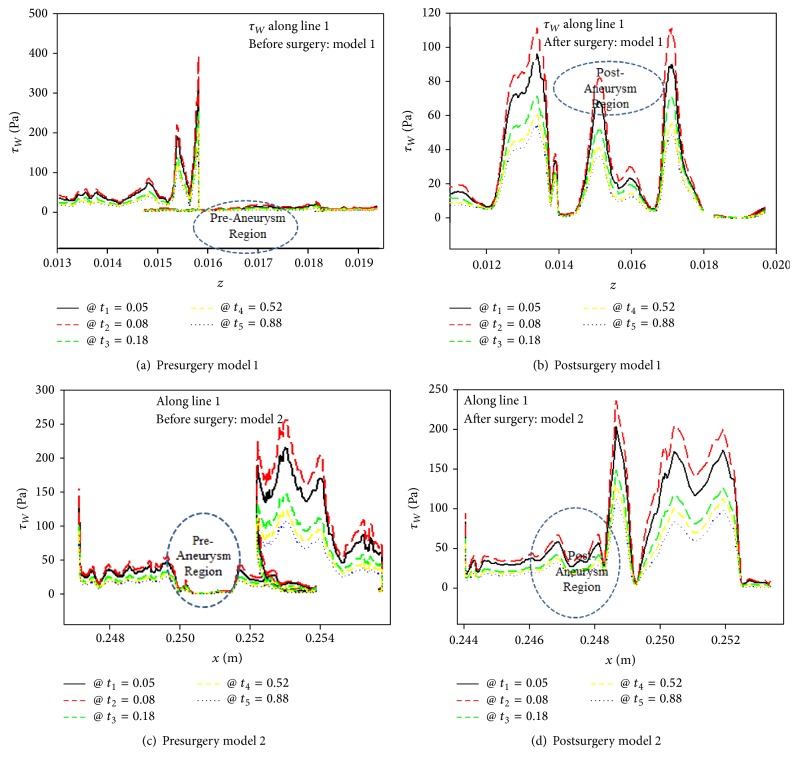
WSS distribution on geometry models 1 and 2 before and after surgery at five different times.

Figure 8 shows the WSS distributions along the yellow lines (Line 1) of preaneurysm and postaneurysm sides at five distinct time points. For preaneurysm model 1, the magnitude of WSS at the throat of superior division of left MCA has the peak value due to the impingement of blood flow into the aneurysm. Inside of the dome, WSS will dramatically drop due to low flow velocity and flow recirculation. The average value of WSS along the dome line has 3.64 Pa for peak flow (at *t*_2_), while the average WSS has 2.14 Pa at *t*_5_. Fukazawa et al. [19] reported that the average WSS at the dome was 2.27 Pa. For postaneurysm model 1, the magnitudes of WSS increase to 43.8 Pa at *t*_2_ and 19.7 Pa at *t*_5_. Previous studies [20, 21] have shown that the magnitudes of WSS in healthy middle cerebral arteries were approximately 18 to 22 Pa. In particular, Lindekleiv et al. [22] found the maximum WSS of 33.17 Pa at the female MCA bifurcation with the blood velocity 0.75 m/s. It was even hard to compare our patient-specific models with Lindekleiv et al. idealized models [22]. The maximum WSS in the MCA was 24% higher in our simulation compared with the result of Lindekleiv et al. [22].

In contrast to model 1, the very low WSS in model 2 of less than 2.5 Pa can be seen in the dome area. The WSS is low due to the slow blood velocity in the dome region, while the blood flow in model 1 is separated in both directions in places where the blood flow momentum can impinge. However, it can be seen that the WSS acting on the blood vessel after operation for model 2 has the maximum 107.36 Pa and the minimum 42.37 Pa. For the comparison of the geometry shapes between models 1 and 2, it can be seen that the WSS in normal blood vessel of model 2 after surgery is about three times larger than that of model 1.

**Figure 9:**
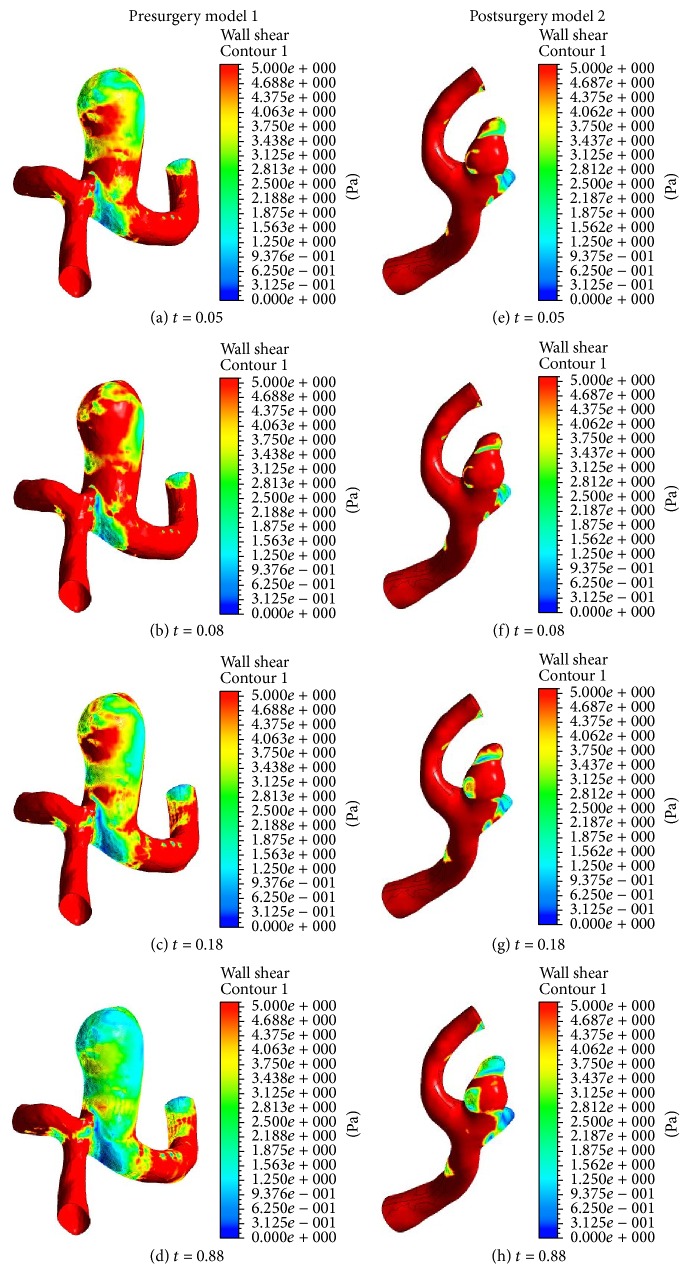
Wall shear stress distributions for models 1 and 2 before surgery at four different times.

In our study the low WSS found in the dome along line 1 in Figure 1 is consistent with CFD study by Shojima et al. [12].  Figure 9 shows the WSS distributions for models 1 and 2 before surgery at four different times. In Figure 9 the WSS in model 1 around the aneurysms surface at peak flow rate, *t* = 0.08, was unevenly distributed. However, the WSS distributions changed considerably with complex flow patterns (see Figure 4(d)) during the diastolic phase. Further, the whole area of aneurysms was exposed to low WSS.

Since the pressure gradient (see Figures 7(a)–7(d)) has been favorable in the left distal vertebral artery, the blood flow in the region is uniform as shown in Figures 5(a)–5(d). As a result, during the whole cardiac cycle the WSS on the left distal vertebral artery is homogeneously distributed in model 2. The heterogeneous distributions of WSS are shown in the aneurysmal regions (see Figures 9(e)–9(h)). As the blood velocity decreases, it can be seen that the regions of low WSS on the daughter saccular aneurysms which is indicated by the red star in presurgery model 2 in Figure 2 widen. It is more likely to rupture the aneurysms in this region.

## 4. Conclusions

The purpose of this study was to investigate the hemodynamic characteristics in the cerebral arteries before and after surgery of the aneurysms under realistic flow conditions. We performed computational fluid dynamic simulations for the geometries generated from CT image-based patient-specific geometries taken before and after surgery. The two patients in the study underwent cerebral angiography with 3D reconstruction using image processing software for 3D design and modeling developed by Materialise. The differences in the unsteady flow, pressure, and WSS between pre- and postaneurysms models were characterized in this study.

Even when the blood vessel geometry was quite different, this study found that the flow patterns with the complex vertical structures inside the aneurysm were similar. The results of flow patterns for models 1 and 2 after the surgery were quite different. It was also discovered that the highest pressure occurred at the distal neck of aneurysms. The average wall shear stress after surgery for model 1 was approximately three times greater than that before surgery, while it was about twenty times greater for model 2. The comparison between models 1 and 2 showed that the WSS in normal blood vessel of model 2 after surgery was about three times larger than that of model 1.

The maximum WSS appeared at the proximal superior division of left MCA in model 1 and at the distal aneurysm neck in model 2. During the diastolic phase the magnitudes and distributions of WSS in aneurysm region in models 1 and 2 were markedly reduced and widen. In particular, the area of low WSS in the daughter saccular aneurysm region in model 2 is associated with aneurysm rupture. Thus the distribution of WSS in aneurysm region provides useful prediction for the risk of aneurysm rupture.

There are limitations to this study. Although the vessel wall is elastic in the study, we do not consider the elasticity of the blood vessel. However, it is believed that the compliance of the blood vessel may have an important role in influencing the hemodynamic changes. The boundary conditions at the inlet and outlet were not patient-specific but were applied from Takao et al. [1]. The study also only looked at two patients for the simulation with the atmospheric pressure at both outlets. With consideration of the elasticity and the real physiological conditions, we expect to perform the simulation for future analysis.
